# 3D4 cells exhibit transcriptional features inconsistent with alveolar macrophage identity

**DOI:** 10.1186/s13567-025-01638-1

**Published:** 2025-10-20

**Authors:** Wenjuan Ma, Frieder Hadlich, Nares Trakooljul, Klaus Wimmers, Eduard Murani

**Affiliations:** Working Group Physiological Genomics, Research Institute for Farm Animal Biology (FBN), Wilhelm-Stahl-Allee 2, 18196 Dummerstorf, Germany

**Keywords:** Porcine alveolar macrophages, 3D4 cell lines, lipopolysaccharide, immunometabolism, transcriptome, Toll-like receptor

## Abstract

The 3D4 cell lines are widely used as porcine alveolar macrophage models in immunological research. However, our preliminary experiments revealed that 3D4/21 cells failed to respond to lipopolysaccharide stimulation. We challenged both 3D4/21 and 3D4/2 clones with various Toll-like receptor (TLR)4 (LPS, Ultrapure LPS, Kdo2-Lipid A) and TLR2/TLR1 (Pam3CSK4) ligands. Strikingly, all stimulants failed to activate pro-inflammatory responses (*IL1B* and *TNF* expression). To clarify the lack of response of 3D4 cells, we isolated primary porcine alveolar macrophages (PAM) from piglets using lung lavage, and analyzed the transcriptome of unstimulated or Kdo2-Lipid A-stimulated 3D4/21 cells compared with equally treated PAM. We identified extensive transcriptomic differences, with 10,718 differentially expressed genes between untreated 3D4/21 cells and PAM (adjusted *p*-value < 0.05). Key alveolar macrophage markers and lineage-determining factors (*SPI1* and *IRF8*) were weakly expressed or absent in 3D4/21 cells. Important pathogen recognition genes (*TLR1*, *TLR2*, *TLR4*, *TLR6*, *TLR7*, *TLR8*, *TLR9*, *CGAS*, *IFI16*, and *NOD2*) showed very low abundance or complete absence compared with PAM. Accordingly, 3D4/21 cells failed to respond to Kdo2-Lipid A stimulation, while PAM showed 2945 regulated genes. Functional annotation revealed that genes preferentially expressed in 3D4/21 cells were enriched for epithelial characteristics. Human Lung Cell Atlas analysis corroborated that 3D4/21 cells originate from respiratory epithelial cells. Similar patterns were observed in 3D4/2 cells, with low macrophage markers (*CD163*, *SPI1*, *CD14*, *TLR2*, and *TLR4*) and high epithelial markers (*TP63*, *EGFR*, and *KRT5*). Our data strongly suggest that 3D4 cell lines were misclassified as myeloid cells and actually possess respiratory epithelial characteristics.

**Supplementary Information:**

The online version contains supplementary material available at 10.1186/s13567-025-01638-1.

## Introduction

The immune response is one of the most important and most intensively researched traits in farm animals because it influences two key aspects of animal production: profitability and animal well-being. A better understanding of the mechanisms of the immune response could help identify trade-offs between production and fitness, and improve disease resilience [1]. Moreover, pigs are becoming increasingly important as animal models in biomedical research because they are more physiologically relevant than traditional rodent models [2–4]. One potential factor in the trade-off between performance and health, which so far has received little attention in farm animals, is the close connection between the activation state of immune cells and their energy metabolism, termed immunometabolism [5, 6]. Cellular in vitro models represent a valuable alternative to in vivo research, especially for the investigation of basic mechanisms and signaling pathways, reducing or even replacing animal experiments. Besides contributing to the implementation of the 3R (replace, reduce, refine) principle in animal research [7], cellular in vitro models have the advantage of a well-controlled and defined environment. Immortalized cell lines provide additional benefit of easier handling and virtually uniform state compared with primary-cell-derived in vitro models [8]. However, there are several issues that limit the utility of cell lines, including misidentification, contamination, and genetic and phenotypic instability [9]. In farm animals, an additional limitation is the paucity of available cell lines. For our research on immunometabolism [6], we were particularly interested in finding a suitable porcine macrophage cell line. Macrophages are cells of the innate immune system that, among many other functions, play a central role in the initiation and resolution of the inflammatory response [10]. Polarization of macrophage activation states into pro-inflammatory (M1) or an anti-inflammatory/resolving (M2) is accompanied by extensive changes in energy metabolism [10, 11]. Alveolar macrophages are the primary targets of several important pathogens in pigs, such as the porcine respiratory and reproductive syndrome virus [12] or African swine virus [13]. Therefore, researchers have developed several immortalized cell lines derived from porcine macrophages, mainly alveolar macrophages [14–16]. Among the most widely used alveolar macrophage lines in pigs are the 3D4 cell lines (also called immortalized porcine alveolar macrophage cells) established by Weingartl et al. [17], consisting of the three clones 3D4/2, 3D4/21, and 3D4/31. In preliminary experiments (not shown), we challenged the clone 3D4/21, which is the most frequently referenced clone of the 3D4 cell line, using lipopolysaccharide (LPS), to induce pro-inflammatory polarization and to investigate the associated metabolic changes. Strikingly, we observed no changes in the expression of pro-inflammatory cytokines. Therefore, in the present study, we used different Toll-like receptor (TLR) ligands to stimulate immune response and analyzed transcriptome of the 3D4/21 cells as well as the 3D4/2 clone to examine the repertoire of their immune signaling and to discover the cause of the failure to respond to TLR stimulation. Our results show that the 3D4 cells do not show the identity of a macrophage cell, including lack of expression of macrophage signature genes and many pattern recognition receptors.

## Materials and methods

### Cell acquisition, culture, and treatment

The 3D4/21 cell line was purchased from LGC Standards (Wesel, Germany) and the 3D4/2 cell line from Hoelzel Biotech (Köln, Germany). Isolation of peripheral blood mononuclear cells was performed as previously described [6, 18]. For the isolation of primary porcine alveolar macrophages (PAM) by bronchoalveolar lavage [19], lungs were collected from young German Landrace piglets (about 12 weeks old, raised in the experimental farm EAS of the FBN) at regular slaughter. The lungs were examined by an authorized veterinarian during meat inspection, and only healthy specimens were taken. The trachea was closed by a clamp to prevent contamination by blood, and the lungs were transferred on vet ice to a cell culture laboratory. In a sterile laminar flow cabinet, the lungs were flushed with ice-cold Dulbecco’s phosphate-buffered saline (DPBS; PAN Biotech, Aidenbach, Germany) three times. The first lavage was discarded, and the fluid from the two next lavages was collected after filtering through a sieve (150 µM; Retch, Haan, Germany). The cells were pelleted by centrifugation of the lavage fluid for 10 min (400 *g* at 4 °C). Red blood cells were removed by Erythrocyte Lysis Buffer (PAN Biotech), followed by a wash with DPBS, and the remaining cells were filtered using MACS^®^ SmartStrainers (100 µm; Miltenyi, Bergisch Gladbach, Germany) to remove cell clumps. Vitality and purity of the isolated cells were checked on a Gallios flow cytometer (Beckman-Coulter, Krefeld, Germany) following staining with propidium iodide and a CD14 antibody (Mouse anti Pig CD14, clone MIL2, Bio-Rad, Feldkirchen, Germany). In total, PAM from six individuals (two male and four female piglets) were used in this study.

For a preliminary test of the response to different TLR ligands, 3D4 cells were seeded into six-well cell culture plates (Faust Lab Science, Klettgau, Germany) at a density of 1.0 × 10^6^ cells/well, and cultured in Roswell Park Memorial Institute (RPMI) medium containing 2 mM l-glutamine (PAN Biotech, Aidenbach, Germany), supplemented with 10% fetal calf serum (PAN Biotech), 1% minimum essential medium (MEM) nonessential amino acids (NEAA) (PAN Biotech), 100 U/mL penicillin/100 µg/mL streptomycin (PAN Biotech), in a 37 °C, 5% CO_2_ environment overnight. On the next day, the cells were stimulated with either 1 µg/mL LPS (*Escherichia coli* O111: B4 serotype; Sigma-Aldrich, Taufkirchen, Germany), 1 µg/mL Ultrapure LPS (*Escherichia coli* O111: B4 serotype; InvivoGen, Toulouse, France), 1 µg/mL Kdo2-Lipid A (KLA; Sigma-Aldrich), or 1 µg/mL Pam3CSK4 (Pam3; InvivoGen), for 3 h and 24 h, respectively. Peripheral blood mononuclear cells (PBMC) were cultured essentially as previously described [6, 18]. Briefly, 3.0 × 10^6^ cells/well were cultured overnight in RPMI medium containing 2 mM of stable glutamine (PAN Biotech), 10% fetal calf serum (PAN Biotech), and 100 U/mL penicillin/100 µg/mL streptomycin (PAN Biotech), and stimulated as described for the 3D4 cells.

For the whole-transcriptome analysis, the 3D4/21 cells were seeded as described above in duplicate or triplicate wells (intra-assay replicates) and stimulated with 100 ng/mL Kdo2-Lipid A for 24 h. The experiment was repeated three times (inter-assay replicates). Freshly isolated PAM were seeded into 12-well cell culture plates (Faust Lab Science) at a density of 1.5 × 10^6^ cells/well, and cultured in RPMI medium containing 2 mM *L*-glutamine, 10% fetal calf serum, and 100 U/mL penicillin/100 µg/mL streptomycin (all media components PAN Biotech). After approximately 2 h, nonadherent cells were removed, and the culture was continued overnight. Of the remaining cells, approximately 90% were CD14 positive. On the next day, the PAM were treated with 100 ng/mL Kdo2-Lipid A for 24 h in triplicate wells. For all treatments, cells cultured in parallel in complete media without stimulants served as controls.

At the end of the indicated treatment period, the cells were lysed in TRI reagent (Sigma-Aldrich) and stored at −80 °C.

### Gene expression profiling

For RNA extraction, cell material from replicated wells (intra-assay replicates) was pooled and processed in TRI reagent according to the manufacturer’s instructions (Sigma-Aldrich). Following TRI reagent extraction, total RNA was purified, including on-column DNase digestion, using the RNA Clean&Concentrator-5 Kit (Zymo Research, Freiburg, Germany). For gene expression profiling using quantitative real-time PCR (qPCR), complementary DNA (cDNA) was synthesized as described previously [6] using 500 ng of the extracted total RNA, 500 ng random hexamers (Promega, Mannheim, Germany), 500 ng of oligo d(T)13 VN, 40 units of RNasin Plus (Promega), and 200 units of SuperScript III reverse transcriptase (ThermoFisher, Darmstadt, Germany). The qPCR reaction mixture consisted of 2 µl cDNA template, 1× LightCycler 480 SYBRplus Green I Master (Roche, Mannheim, Germany), and primers as presented in Additional File 1. The qPCR reaction was performed in duplicate on a LightCycler 480 System. Besides the genes of interest, *RPL32* was quantified as a normalizing reference gene. Standard curves were obtained by the amplification of a serial dilution of a gene-specific PCR fragment, which was generated for each profiled gene. For samples where no amplification occurred, the crossing point (Cp) was set to 45. Samples with a Cp greater than 35 (the average Cp of the lowest standard with 10 copies was 34) were considered not to express the profiled gene.

For whole-transcriptome analysis, messenger RNA (mRNA) sequencing was performed by Novogene (Cambridge, UK). This included library preparation (directional mRNA library using poly A enrichment) and paired-end sequencing for 2 × 150 bp on a NovaSeq X Plus (Illumina; San Diego, CA, USA). The quality of the analyzed RNA was determined on an Agilent 5400 Bioanalyzer, which showed RNA integrity number (RIN) > 9 for all samples.

### Data analysis

Data preprocessing, including data quality control and filtering, was performed using in-house Perl scripts by Novogene. The sequencing produced on average about 45 Mio clean reads per sample with mean Q30 of 91%. Clean reads were mapped against the reference genome (Ssscrofa11.1, ENSEMBL release 111) using HISAT2 v2.0.5. Uniquely mapped reads (on average 89.9%) were assigned to genomic features and counted using HTSeq version 2.0.2. On the basis of the count data, differential gene expression was analyzed using the DESeq2 software package. Firstly, genes with low expression were removed and only genes with more than ten counts in at least three samples were retained, leaving a total of 14 852 genes. The data were adjusted for the effect of sex. Gene expression was compared between the different cell types in different treatments by calculating linear contrasts, and the resulting *p*-values were adjusted via the Benjamini–Hochberg (BH) procedure. For principal component analysis, data were transformed by means of variance-stabilizing transformation in DESeq2.

For the cell identity analysis, the gene annotation was first updated using data from other ENSEMBL releases (105–113). The Human Lung Cell Atlas version 2 (core) [20] data were obtained from CZ CELLxGENE collections, and filtered (filters: including “lung parenchyma”, and removing subject_type “alive_disease”). Subsequently, the PAM and 3D4/21 transcriptome data were integrated with the filtered Human Lung Cell Atlas data using anchor-based integration pipeline implemented in SEURAT v5. After normalization, the top 2000 highly variable features were selected for subsequent anchor identification. Uniform manifold approximation and projection (UMAP) visualization was performed using the first 50 principal components computed from the integrated dataset with resolution set to 0.8.

Volcano plots of differential expression were drawn using the EnhancedVolcano R package. Heatmaps of gene expression level were generated in GraphPad Prism (version 9.2.0, GraphPad software, San Diego, CA, USA). For functional annotation, the ToppFun option of the ToppGene Suite [21] was employed. Enriched functional terms were summarized and visualized using the REVIGO tool [22].

## Results

### 3D4/21 and 3D4/2 cells show no pro-inflammatory response to TLR4 or TLR2/TLR1 activation

**Figure 1:**
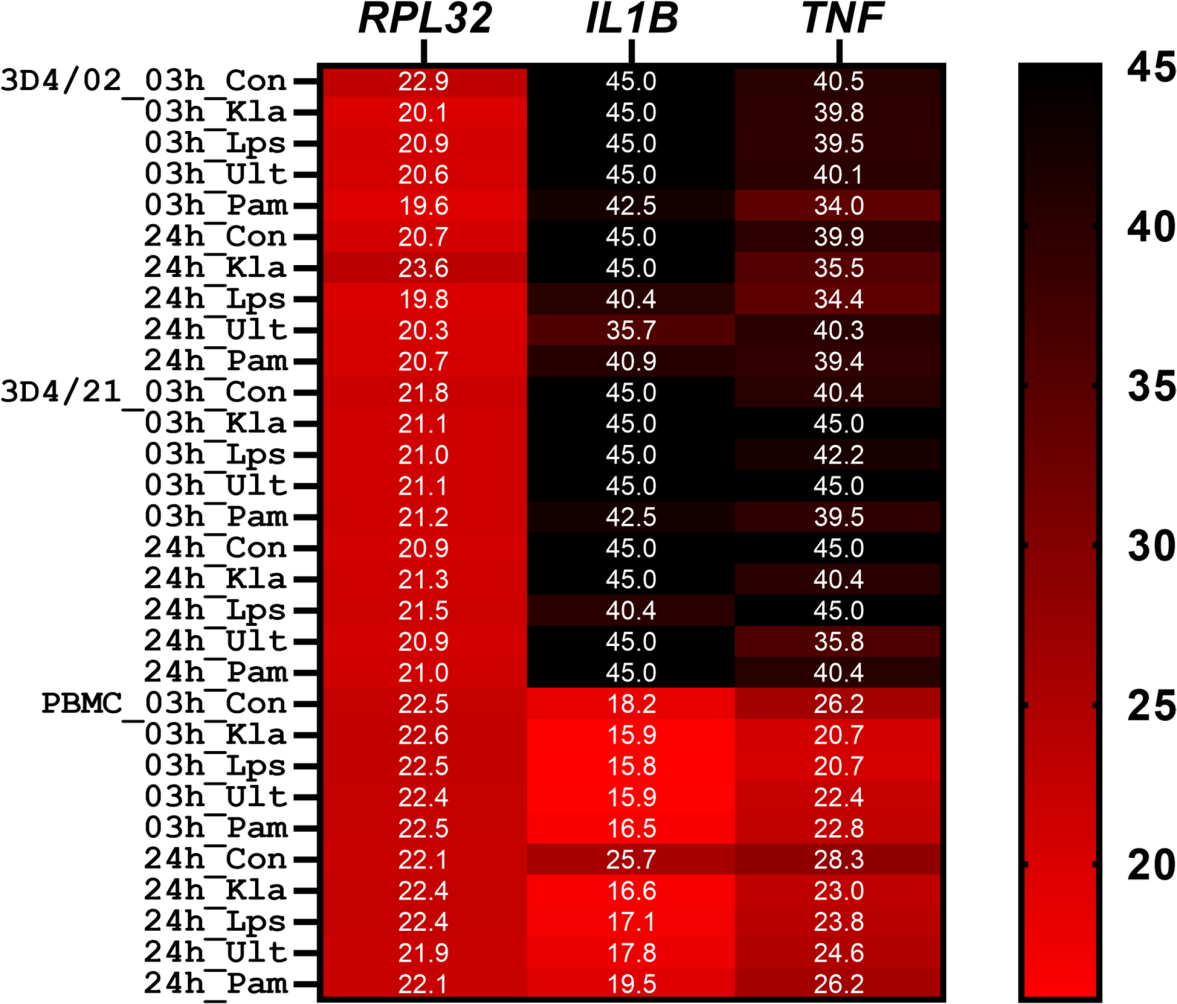
**TLR2 and TLR4 ligands fail to stimulate pro-inflammatory response in 3D4 cells.** Heatmap showing the expression of pro-inflammatory cytokines *IL1B* and *TNF* and the reference gene *RPL32*, after treatment using 1 µg/mL Kdo2-Lipid A (KLA), 1 µg/mL lipopolysaccharide (LPS), 1 µg/mL Ultrapure LPS (Ult), or 1 µg/mL Pam3CSK4 (Pam3), or no stimulant (Con) for 3 h and 24 h, respectively, in 3D4/2 and 3D4/21 cells, and PBMC. The numbers in the heatmap show crossing points (i.e., the cycle threshold, Ct) of quantitative real-time PCR.

In order to verify the lack of response of 3D4/21 cells to LPS from *Escherichia coli* (activates TLR4 and TLR2/TLR1), we stimulated the cells with a variety of TLR4 and TLR2/TLR1 ligands, including Ultrapure LPS (TLR4 ligand), Kdo2-Lipid A (TLR4 ligand), and Pam3CSK4 (TLR2/TLR1 ligand), in addition to LPS. Moreover, we included porcine PBMCs as a positive control, and the 3D4/2 clone to check whether the lack of response is common to the 3D4 cell lineage. As shown in Figure 1, as expected, in PBMCs, all ligands increased the expression of pro-inflammatory cytokines *IL1B* and *TNF* after 3 h as well as 24 h of stimulation. In contrast, expression of *IL1B* and *TNF* was not detectable in either unstimulated or stimulated 3D4/21 and 3D4/2 cells at any time point. These results strongly indicated a deficiency in pathogen recognition by 3D4 cells.

### Transcriptome analysis reveals extensive differences in gene expression between 3D4/21 cells and alveolar macrophages

**Figure 2:**
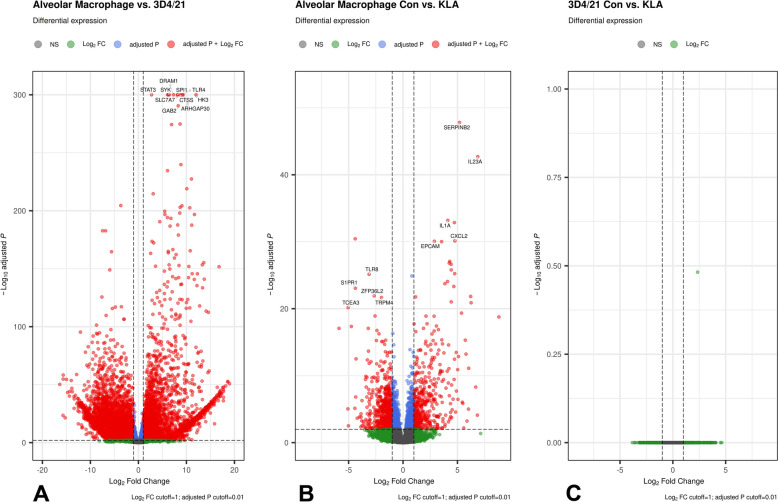
**Transcriptome of 3D4/21 cells shows extensive differences compared with primary porcine alveolar macrophages (PAM).** **A** Volcano plot depicting differential gene expression between untreated 3D4/21 cells and PAM. **B** Volcano plot depicting differential gene expression in PAM after 24 h stimulation using 100 ng/mL Kdo2-Lipid A (KLA) compared with untreated PAM (Con). **C** Volcano plot depicting differential gene expression in 3D4/21 cells after 24 h stimulation using 100 ng/mL Kdo2-Lipid A (KLA) compared with untreated 3D4/21 cells (Con).

When comparing the baseline gene expression between unchallenged 3D4/21 cells and PAM, which should represent the original cell type of 3D4 cells, of the 14 852 expressed genes included in the analysis, 10 718 showed significant differences (adjusted *p*-value < 0.05; Additional file 2). The volcano plot in Figure 2A illustrates the extensive transcriptome differences between the immortalized and primary cells. As further shown in Figure 2A, *TLR4* is one of the most significantly differentially expressed genes (DEGs). Another notable top DEG is *SPI1*, encoding PU.1, a transcription factor that plays a key role in macrophage differentiation (reviewed in [23]). In terms of fold change differences, *CD163*, expressed exclusively in the monocyte–macrophage lineage [24], was one of the top genes upregulated in PAM compared with 3D4/21, where it was completely absent (zero counts).

Functional enrichment analysis of the top 500 genes preferentially expressed in PAM revealed mostly immune response-related functional terms (e.g., GO:0002684: regulation of immune system process) (Additional file 3 and Additional file 4). Remarkably, the top significantly upregulated genes in PAM were enriched for putative PU.1 targets (Additional file 3). In contrast, functional annotation of the top 500 genes preferentially expressed in 3D4/21 cells indicated mainly development-related terms (such as GO:0060429: epithelium development) (Additional file 3 and Additional file 5). Moreover, whereas in the Cell Atlas category lung myeloid cell patterns were enriched for genes preferentially expressed in PAM, for DEGs upregulated in 3D4/21 cells airway epithelial cell related patterns emerged (Additional file 3).

When looking at the genomic distribution of the DEGs, several clusters are evident (Additional file 6). For instance, a C-type lectin/C-type lectin-like domain cluster on chromosome 5 or a zinc finger (ZNF) gene cluster on chromosome 6 are expressed in PAM but are essentially not expressed in 3D4/21 cells. In 3D4/21 cells in turn, all homeobox gene clusters were upregulated compared with PAM.

Taken together, these results showed extensive differences between the transcriptomes of 3D4/21 cells and alveolar macrophages, particularly of immune-response related genes.

### The transcriptome of the 3D4 cells shows similarities to that of lung epithelial cells

Because we found several macrophage lineage-specific marker genes among the top DEGs, we checked the expression of additional markers of alveolar macrophages listed in the Tabula Sapiens human cell atlas [25]. Of the 24 OnClass Marker Genes [26], 19 were present in our genes list (including besides *CD163* and *SPI1* also *C1QB*, *C1QA*, *MRC1*, *C1QC*, *MSR1*, *VSIG4*, *MS4A7*, *MARCO*, *SLCO2B1*, *TYROBP*, *FCER1G*, *LYZ*, *AIF1*, *C5AR1*, *SLC11A1*, *BCL2A1*, and *CD14*). All marker genes featured significantly higher expression in PAM compared with 3D4/21 cells (Additional file 2). Besides *CD163*, an additional eight marker genes of alveolar macrophages (*C1QB*, *C1QA*, *MRC1*, *MSR1*, *VSIG4*, *MS4A7*, *MARCO*, and *SLC11A1*) were completely absent in 3D4/21 cells.

**Figure 3:**
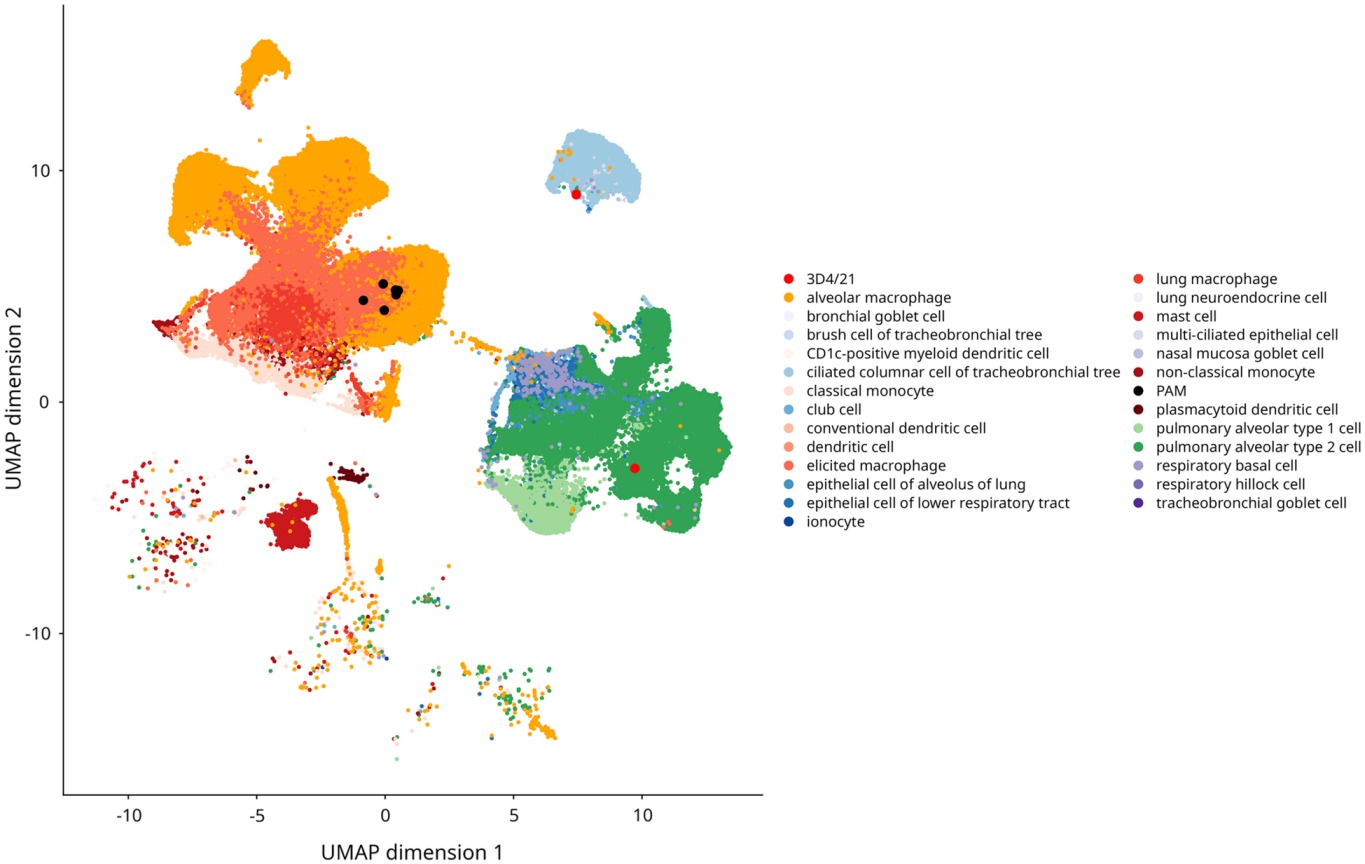
**Transcriptome-based cell type assignment indicates that the 3D4/21 cells have a respiratory epithelial character.** UMAP plot of primary porcine alveolar macrophage (PAM) and 3D4/21 transcriptomes integrated with myeloid and epithelial cells from the Human Lung Cell Atlas version 2 shows that PAM cluster with lung macrophages but 3D4/21 cells cluster with respiratory epithelial cells. PAM and 3D4/21 cells are highlighted by larger dots colored in black and red, respectively.

Together with the results of the functional enrichment analyses, which revealed enrichment of patterns related to epithelial cells in 3D4/21 cells, these findings raised questions about the cell type identity of the 3D4 cells. Therefore, we attempted to determine the cell type of 3D4/21 cells using data from the human lung cell atlas. In a preliminary analysis, we used annotation level 2 to reduce the number of cell types/categories for a better clarity. This analysis confirmed myeloid identity of PAM, and supported the evidence for the epithelial-like identity of the 3D4/21 cells (Additional file 7). Because 3D4/21 cells grow continuously, we hypothesized that they might represent respiratory epithelial stem cells. In fact, the 3D4/21 cells show significantly higher abundance (Additional file 2) of several marker genes (such as *EGFR*, *TP63*, and *KRT5*) of respiratory basal cells [27, 28]. In contrast, most markers of pulmonary alveolar type 2 cells (AT2), bronchioalveolar stem cells, or club cells [29–31] were either absent in both 3D4/21 cells and PAM (e.g., *SFTPC*, *SFTPB*, *SFTPA*, *SCGB1A1*, and *SCGB3A2*) or did not show higher abundance in 3D4/21 cells (*ABCA3* and *ETV5*). However, when identity assignment was focused on lung myeloid and epithelial cells, the 3D4/21 cells did not unambiguously cluster with any specific cell type, including respiratory basal cells (Figure 3). On the other hand, this analysis confirmed our PAM cells as lung macrophages.

**Figure 4:**
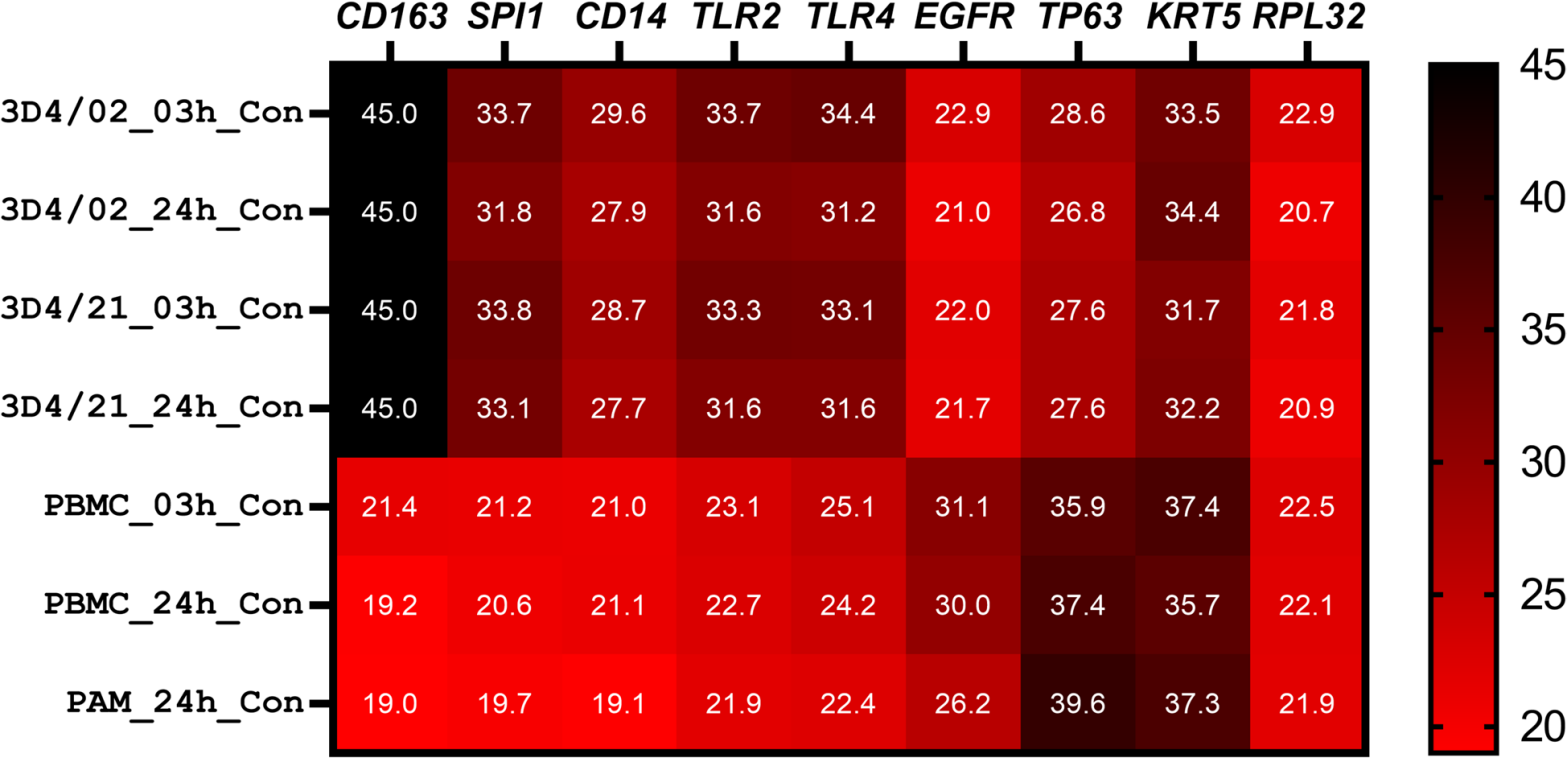
**3D4/21 and 3D4/2 cells show a similar pattern of the expression of macrophage and respiratory epithelial stem cell marker genes.** Heatmap showing the expression of macrophage (*CD163*, *SPI1*, *CD14*, *TLR2*, and *TLR4*) and respiratory epithelial stem cell marker genes (*EGFR*, *TP63*, and *KRT5*) in untreated 3D4/2, 3D4/21, PBMC, and primary porcine alveolar macrophages at different time points. The numbers in the heatmap show crossing points (i.e., the cycle threshold, Ct) of quantitative real-time PCR.

We analyzed the expression of several marker genes of alveolar macrophages, pattern recognition receptors (PRRs), as well as marker genes of respiratory basal cells in 3D4/2 cells using qPCR. This analysis showed similar expression profile of 3D4/2 cells compared with 3D4/21 cells (Figure 4).

Taken together, this analysis strengthens the evidence for a nonmyeloid, epithelial-like transcriptional signature of 3D4 cell, but does not provide definitive assignment to a specific cell type.

### 3D4/21 cells show low abundance of several pattern-recognition-related genes

Stimulation of PAM for 24 h with Kdo2-Lipid A significantly (adjusted *p*-value < 0.05) influenced the expression of 2945 genes (Figure 2B). This included the expected upregulation of pro-inflammatory cytokines such as *IL1A* (Figure 2B), *IL1B*, and *TNF* (Additional file 2). Accordingly, the DEGs are enriched for immune response-related functional terms (such as GO:0006954: inflammatory response) (Additional file 3 and Additional file 8). In contrast, as shown in Figure 2c, the 3D4/21 cells did not show any response at all to Kdo2-Lipid A (Additional file 2). This result confirms the initial qPCR experiment using different TLR ligands. The baseline and treatment-induced transcriptome differences are reflected in the PCA plot, where the cells are clearly separated by type (PAM versus 3D4) and treatment (unstimulated versus stimulated) (Additional file 9).

**Figure 5:**
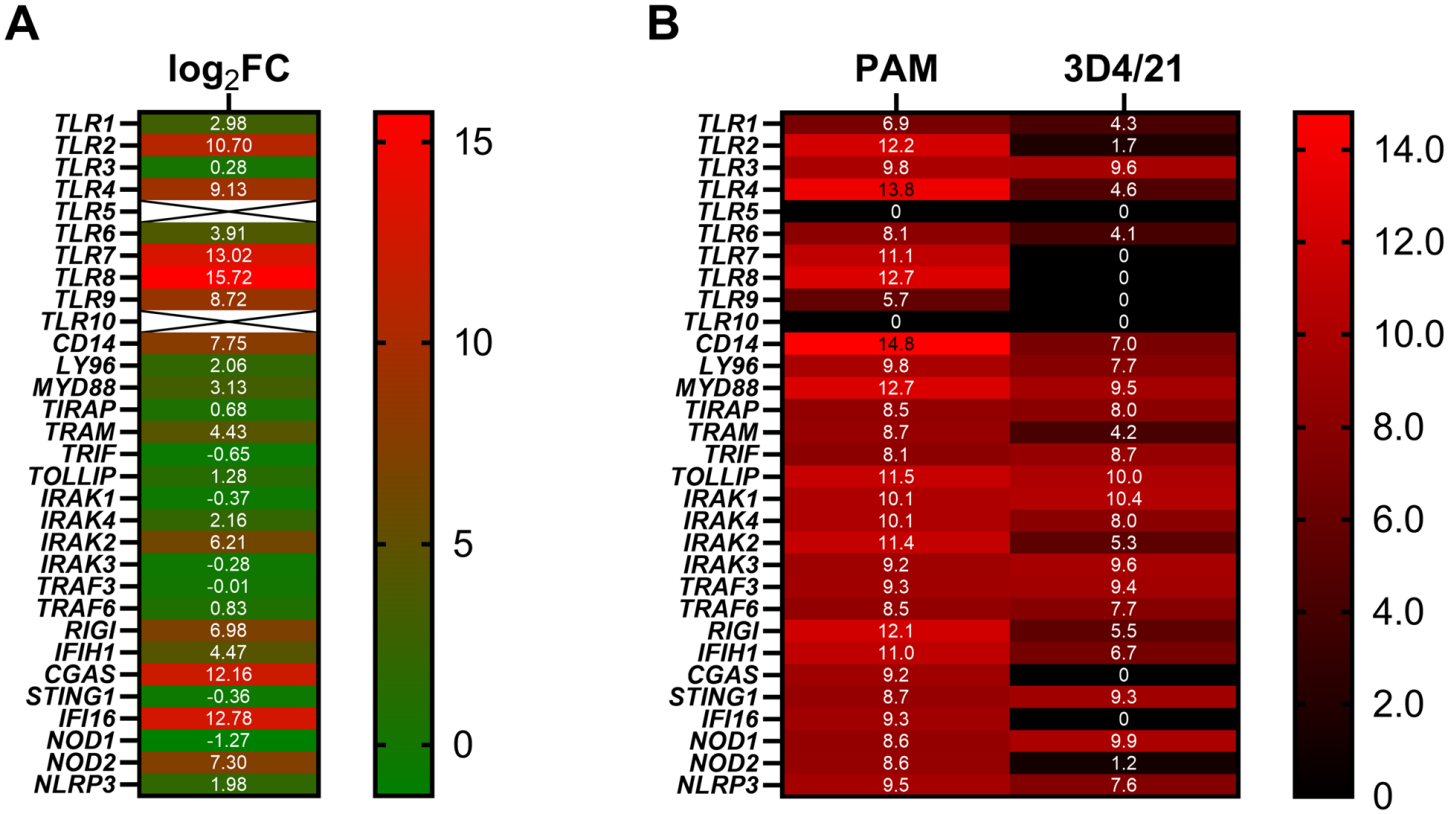
**3D4/21 cells show low abundance of several pattern recognition-related genes**. **A** Log_2_ fold-change differences in the expression of the main PRR-related genes between untreated 3D4/21 cells and primary porcine alveolar macrophages obtained from mRNA-sequencing data. **B** Heatmap of the abundance of the main PRR-related genes between untreated 3D4/21 cells and porcine alveolar macrophages obtained from mRNA-sequencing data. The numbers indicate average log_2_-transformed normalized counts.

Because the 3D4/21 cells failed to respond to diverse TLR stimuli, we took a closer look at the expression of a panel of PRRs and associated signaling molecules. As depicted in Figure 5, several PRR genes showed much lower expression in 3D4/21 cells compared with PAMs, particularly *TLR2*, *TLR4*, *TLR7*, and *TLR8*. Further, as shown in Figure 4, qPCR analysis revealed similarly low levels of the expression of *TLR2*, *TLR4*, and *CD14* in 3D4/2 cells. Therefore, these results provide an explanation for the lack of response to TLR4 and TLR2/TLR1 ligands in 3D4 cells, and indicate that the 3D4 cells are not an appropriate in vitro cellular model to study porcine immune responses to bacteria.

## Discussion

In the present study, we characterized 3D4 cells in terms of their identity and responsiveness to pathogen-associated molecular patterns by examining their transcriptome. Previously, Li et al. [32] investigated structural variation in the genome of 3D4/21 cells. The authors included a comparison of the transcriptome of unstimulated 3D4/21 cells with that of PAM. Unfortunately, the authors did not publish the original data, and did not present the results of the comparison of the whole transcriptome, only the top 20 DEGs preferentially expressed in either cell type. Nevertheless, the presented DEGs show a high concordance with our results, including several marker genes of alveolar macrophages being inactive in 3D4/21 cells, such as *CD163*, *C1QC*, and *MRC1*. Other authors also reported low abundance of macrophage-specific markers such as CD14, SWC3 (encoded by *SIRPA*), and CD169 in 3D4 cells [13, 17], which is in line with our results (Additional file 2). Further, our results confirm dysregulation of gene expression in several regions of structural variation reported by Li et al. [32], especially in the area of large deletions on chromosomes Ssc1, Ssc5, and Ssc10 (Additional file 2). Our results revealed marked downregulation of *SPI1* expression in 3D4 cells, including both the 3D4/21 and 3D4/2 clones. The transcription factor PU.1 encoded by *SPI1* plays a crucial role in differentiation, polarization, and survival of macrophages [23]. PU.1 governs macrophage-specific gene expression, including *TLR4*, whose expression in myeloid cells is driven by a conserved PU.1-dependent proximal promoter [33]. Interestingly, an additional five genes downstream of *SPI1* on chromosome Ssc2 also show more or less dramatic downregulation in 3D4/21 cells compared with PAM (Additional file 2). Li et al. [23] did not report any structural variation in this region, so the cause for the downregulation of *SPI1* and the neighboring genes in 3D4/21 cells remains unclear. To regulate the macrophage-specific gene repertoire, PU.1 interacts with partner transcription factors, such as interferon regulatory factor 8 (IRF8), that critically contribute to its recruitment to genomic binding sites [34]. IRF8 binds DNA constitutively with PU.1, establishing baseline expression of genes related to basal macrophage functions, antigen presentation, cell adhesion, and lymphocyte activation molecules [34, 35]. The loss of IRF8 leads among others to defective expression of pathogen-associated molecular pattern receptors [35]. We found *IRF8* to be essentially transcriptionally inactive in 3D4/21 cells (Additional file 2). Additional important lineage determining transcription factors [36] that are essentially absent in 3D4/21 cells are *MAFB* and *CEBPA* (Additional file 2). None of these genes lies in area of structural variation reported by Li et al. [32]. Taken together, the absence of several vital lineage determining transcription factors could explain the loss of macrophage transcriptional signature in 3D4/21 cells. Yet, when performing functional annotation of genes preferentially expressed in 3D4/21 cells and cell-type assignment, we found striking similarities with respiratory epithelial cells. In addition, 3D4/2 cells exhibit a similar profile of marker genes to 3D4/21 cells, including upregulation of selected marker genes of respiratory epithelial stem cells and downregulation of canonical macrophage markers compared with PAM. These findings indicate that misclassification of 3D4 cells is more likely than spontaneous change caused by a loss of the macrophage lineage-determining factors. In fact, different cell identity could explain the extensive transcriptome differences between 3D4 cells and PAM. Unfortunately, we were unable to definitively determine the respiratory epithelial cell type of the 3D4/21 cells. Owing to their similarity to pulmonary alveolar type 2 cells at the transcriptome level, as well as their expression of *TP63* and *KRT5*, we speculate that 3D4 cells could represent multipotent distal airway stem cells. These rare cells are positive for *TP63* and *KRT5* and able to differentiate into AT2 cells following injury [37]. Notably, Weingartl et al. [17] reported that 3D4 cells do not express SV40 large T-antigen and suspected an alternative mechanism for their continuous growth. Our hypothesis is that the continuous growth of 3D4 cells is driven by their stem cell identity. In fact, besides *TP63*, the 3D4/21 cells show high abundance of several other stemness markers [38, 39] such as *MYC*, *FOXM1*, and *YAP1* (Additional file 2). Moreover, it is of note that Li et al. [32] did not report large chromosomal aberrations that are typical for immortalized cells [40]. Respiratory epithelial cells play an important role in defending the respiratory tract against pathogens. They do so not only passively through their barrier function, but also actively by sensing pathogens and inducing an immune response [27, 38, 41]. Respiratory tract disorders, including infectious diseases, are a major cause of morbidity and mortality in pigs as well as in humans. Unlike in rodents, the respiratory epithelium of pigs shows resemblance to humans [42]. Accordingly, pigs are becoming increasingly relevant as model animals for human respiratory disorders such as cystic fibrosis [42, 43]. This has led to a surge in interest in in vitro culture of porcine respiratory epithelial cells [42]. We found that 3D4/21 cells express high levels of *TLR3*, consistent with the evidence suggesting their respiratory epithelial origin, as TLR3 is the most abundant Toll-like receptor in these cells [44]. Most common respiratory viruses replicate primarily in respiratory epithelial cells [45]. This could explain the susceptibility of the 3D4 cells to different viruses [17]. Furthermore, 3D4/21 cells express key cytokines and chemokines produced by respiratory epithelial cells in response to viral infection/TLR3 activation [46], including *IL6*, *IL8*, and *CCL2* (Additional file 2). Overall, the repertoire of pattern recognition-related genes expressed in 3D4/21 cells suggests that they are more sensitive to RNA viruses but less sensitive to DNA viruses (indicated by the absence of DNA sensors *IFI16* and *CGAS*). In fact, RNA viruses play a more important role in respiratory tract diseases [45]. In conclusion, our findings show that the transcriptional profile of 3D4 cells does not match the expected pattern of myeloid cells. Instead, our findings provide convincing evidence that 3D4 cells in fact originate from respiratory epithelial cells. This discovery highlights the crucial need for comprehensive characterization of commonly used cell lines using omics tools [47]. Our findings establish a knowledge base for the further use of 3D4 cell lines in immunological research in pigs.

## Supplementary Information

**Additional file 1.** **Oligonucleotides used in qPCR.**

**Additional file 2. Lists of differentially expressed genes.**

**Additional file 3. Functional enrichment analysis, transcriptional regulators, and co-regulated cell types of differentially expressed genes in PAM vs. 3D4/21 cells and KLA treatment responses.**

**Additional file 4. Functional enrichment of top 500 genes preferentially expressed in primary porcine alveolar macrophages (PAM) compared to 3D4/21 cells under baseline conditions.**

**Additional file 5. Functional enrichment of top 500 genes preferentially expressed in 3D4/21 cells compared to primary porcine alveolar macrophages (PAM) under baseline conditions.**

**Additional file 6. Genome distribution of differentially expressed genes between primary porcine alveolar macrophages and 3D4/21 cells under baseline conditions.**

**Additional file 7. Preliminary transcriptome-based cell type assignment of the 3D4/21 cells and of primary porcine alveolar macrophages (PAM).**

**Additional file 8. Functional enrichment of differentially expressed genes (adjusted* p*-value < 0.05) following 24 h stimulation of primary porcine alveolar macrophages (PAM) using 100 ng/mL Kdo2-Lipid A (KLA). **

**Additional file 9. Principal component analysis of 3D4/21 cells and of primary porcine alveolar macrophages (PAM) based on transcriptome data obtained in unstimulated conditions (Con) or after 24h stimulation using 100 ng/mL Kdo2-Lipid A (KLA).**

## Data Availability

The datasets used and analyzed during the current study are available from the corresponding author upon reasonable request.
