# Supplementary material for: 3D4 cells exhibit transcriptional features inconsistent with alveolar macrophage identity
Source: Vet Res. 2025 Oct 20;56:201. doi: 10.1186/s13567-025-01638-1 (PMC12539023; doi:10.1186/s13567-025-01638-1)
Supplement: Supplementary file 4 — Additional file 4. Functional enrichment of top 500 genes preferentially expressed in primary porcine alveolar macrophages (PAM) compared to 3D4/21 cells under baseline conditions. [file 13567_2025_1638_MOESM4_ESM.docx]

**
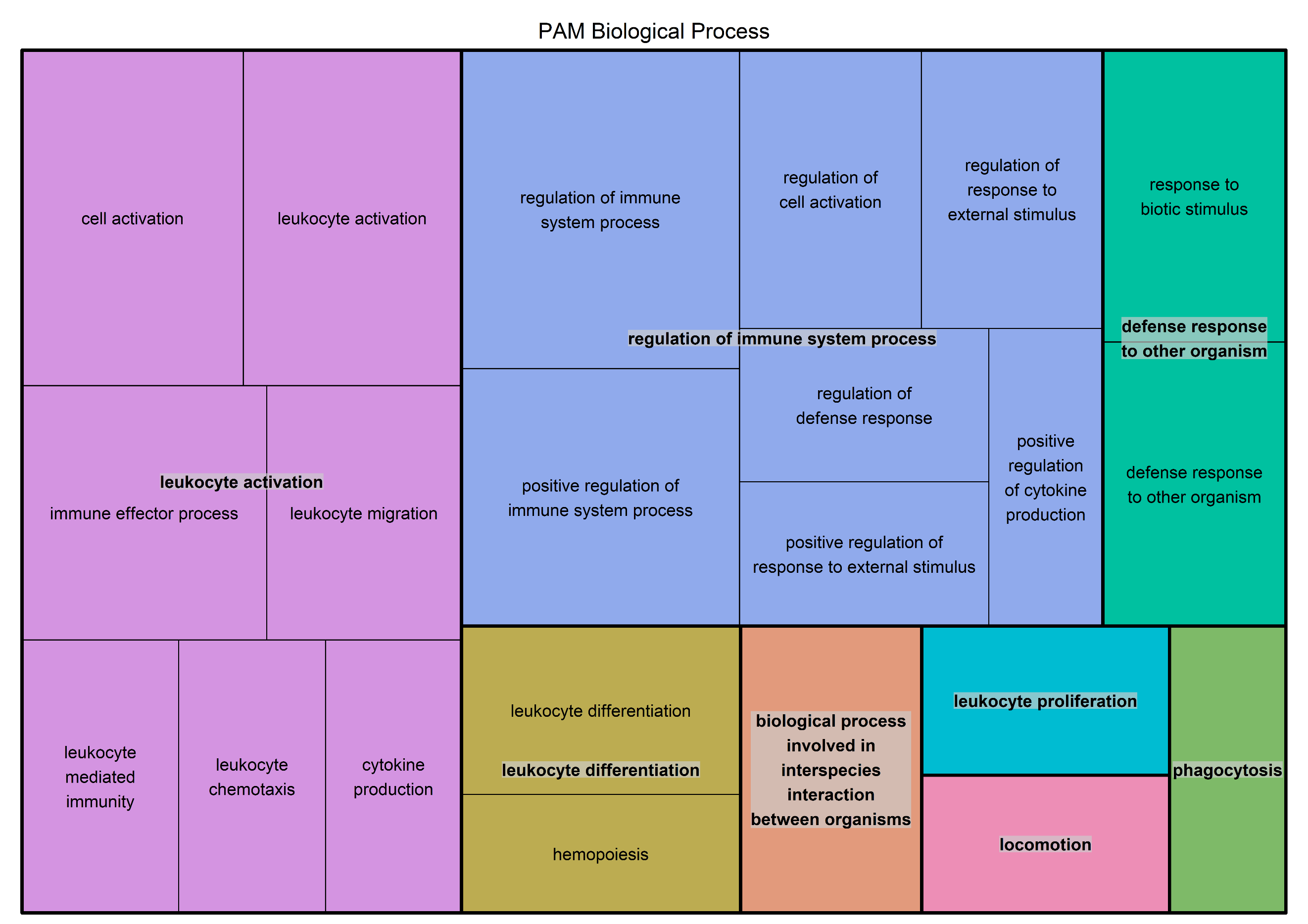
**

**Additional file 4.** **Functional enrichment of top 500 genes preferentially expressed in primary porcine alveolar macrophages (PAM) compared to 3D4/21 cells under baseline conditions.** The tree map generated by REVIGO shows a summary of enriched GO terms related to biological processes.
