# Supplementary material for: 3D4 cells exhibit transcriptional features inconsistent with alveolar macrophage identity
Source: Vet Res. 2025 Oct 20;56:201. doi: 10.1186/s13567-025-01638-1 (PMC12539023; doi:10.1186/s13567-025-01638-1)
Supplement: Supplementary file 6 — Additional file 6. Genome distribution of differentially expressed genes between primary porcine alveolar macrophages and 3D4/21 cells under baseline conditions. [file 13567_2025_1638_MOESM6_ESM.docx]

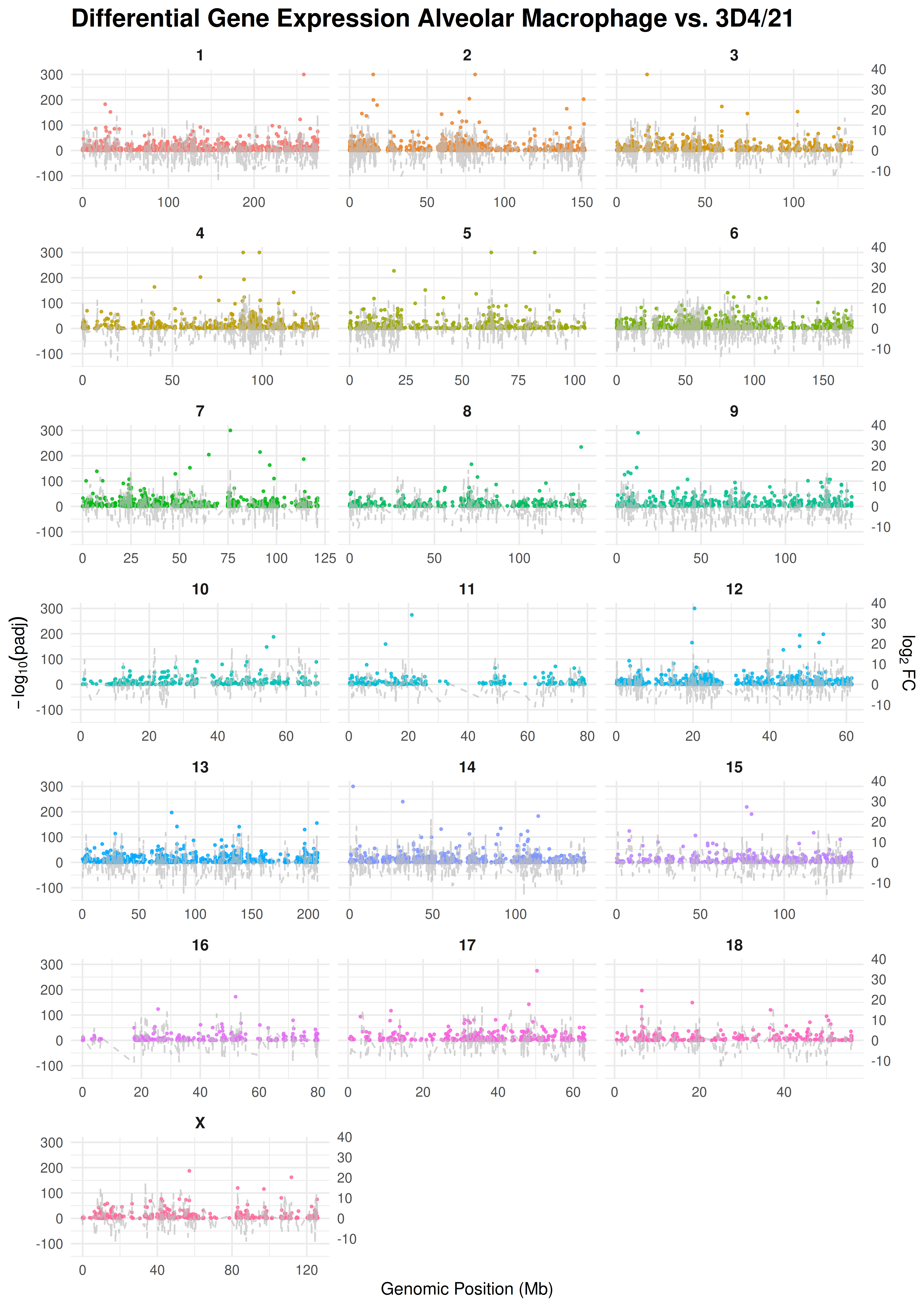


**Additional file 6.** **Genome distribution of differentially expressed genes between primary porcine alveolar macrophages and 3D4/21 cells baseline conditions.** The coloured dots represent -log_10_ of the adjusted p-value (padj) for differential expression (left y-axis). The gray dashed line represents log_2_ fold changes (FC) between primary porcine alveolar macrophages and 3D4/21 cells (right y-axis). Chromosome number is on top of each graph and chromosome coordinates in Megabases are plotted on the x-axis.
