# Supplementary material for: 3D4 cells exhibit transcriptional features inconsistent with alveolar macrophage identity
Source: Vet Res. 2025 Oct 20;56:201. doi: 10.1186/s13567-025-01638-1 (PMC12539023; doi:10.1186/s13567-025-01638-1)
Supplement: Supplementary file 7 — Additional file 7. Preliminary transcriptome-based cell type assignment of the 3D4/21 cells and of primary porcine alveolar macrophages (PAM). [file 13567_2025_1638_MOESM7_ESM.docx]

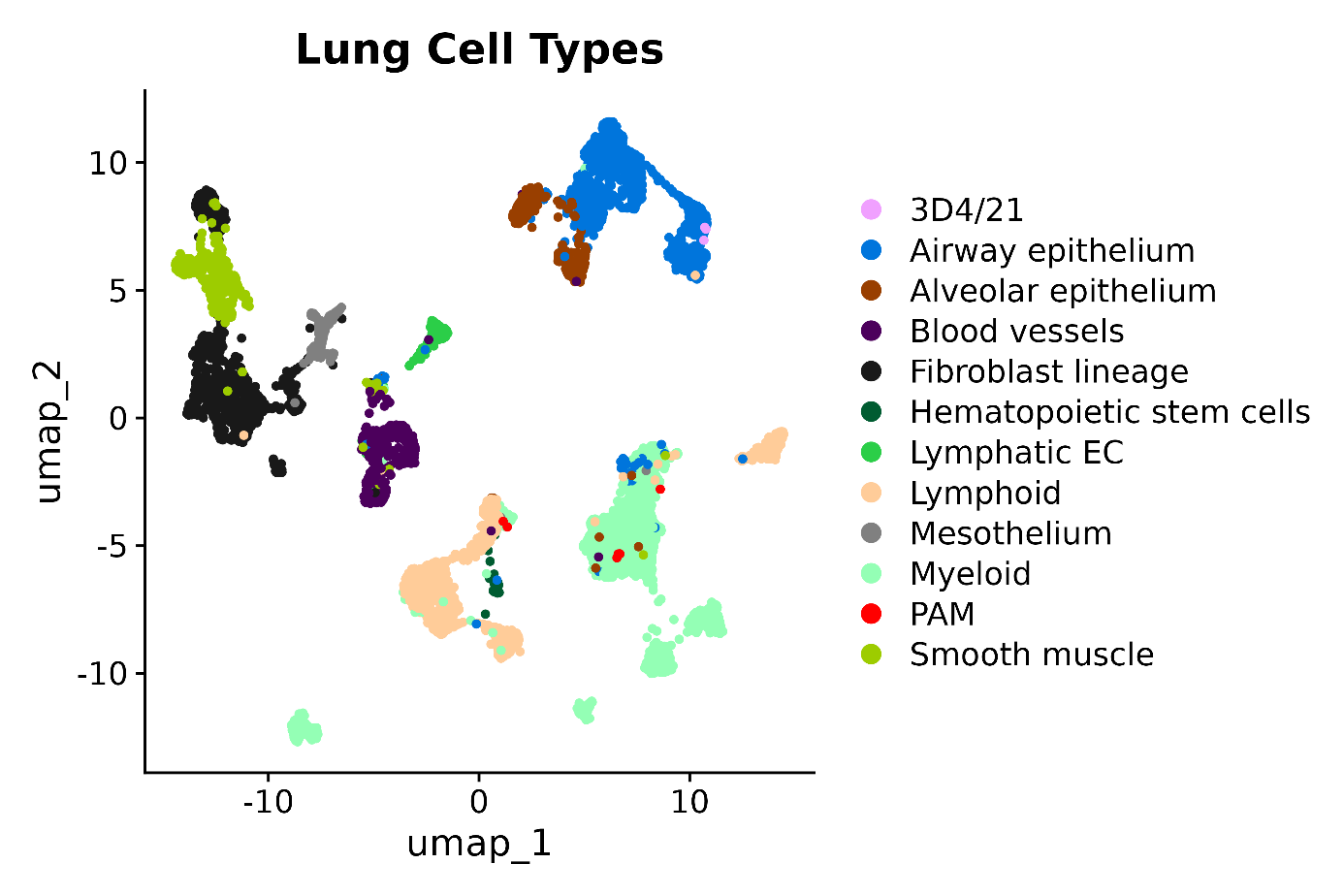


**Additional file 7. Preliminary transcriptome-based cell type assignment of the 3D4/21 cells and of primary porcine alveolar macrophages (PAM).** UMAP plot of primary porcine alveolar macrophage (PAM) and 3D4/21 transcriptomes integrated with data from the Human Lung Cell Atlas v2. Cell types of Human Lung Cell Atlas v2 are annotated by the level 2 cell-type information.
