# Supplementary material for: 3D4 cells exhibit transcriptional features inconsistent with alveolar macrophage identity
Source: Vet Res. 2025 Oct 20;56:201. doi: 10.1186/s13567-025-01638-1 (PMC12539023; doi:10.1186/s13567-025-01638-1)
Supplement: Supplementary file 8 — Additional file 8. Functional enrichment of differentially expressed genes (adjusted p-value < 0.05) following 24 h stimulation of primary porcine alveolar macrophages (PAM) using 100 ng/mL Kdo2-Lipid A (KLA). [file 13567_2025_1638_MOESM8_ESM.docx]

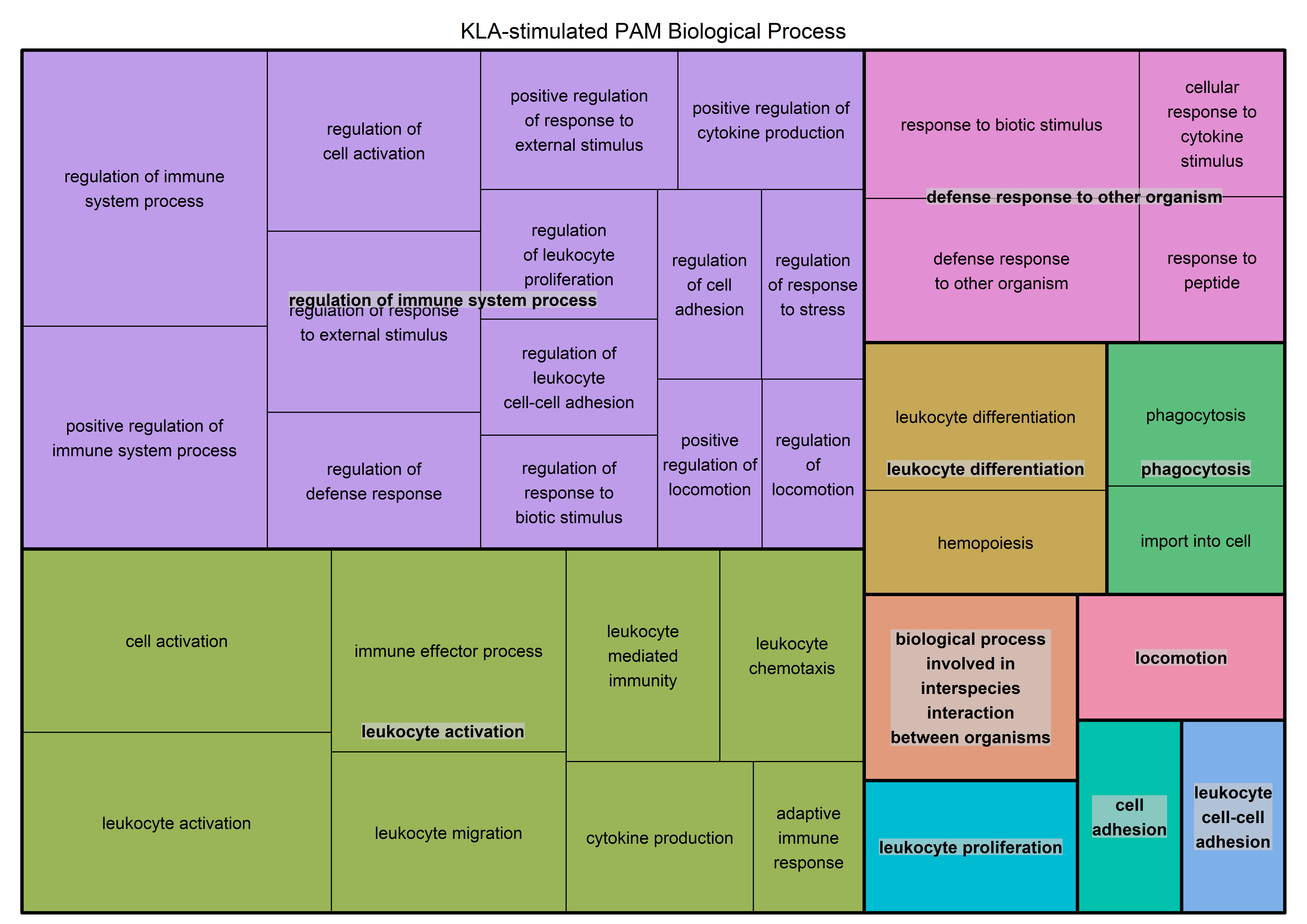


**Additional file 8. Functional enrichment of differentially expressed genes (adjusted p-value<0.05) following 24h stimulation of primary porcine alveolar macrophages (PAM) using 100 ng/mL Kdo2-Lipid A (KLA).** The tree map generated by REVIGO shows a summary of enriched GO terms related to biological processes.
