# Supplementary material for: 3D4 cells exhibit transcriptional features inconsistent with alveolar macrophage identity
Source: Vet Res. 2025 Oct 20;56:201. doi: 10.1186/s13567-025-01638-1 (PMC12539023; doi:10.1186/s13567-025-01638-1)
Supplement: Supplementary file 9 — Additional file 9. Principal component analysis of 3D4/21 cells and of primary porcine alveolar macrophages (PAM) based on transcriptome data obtained in unstimulated conditions (Con) or after 24h stimulation using 100 ng/mL Kdo2-Lipid A (KLA). [file 13567_2025_1638_MOESM9_ESM.docx]

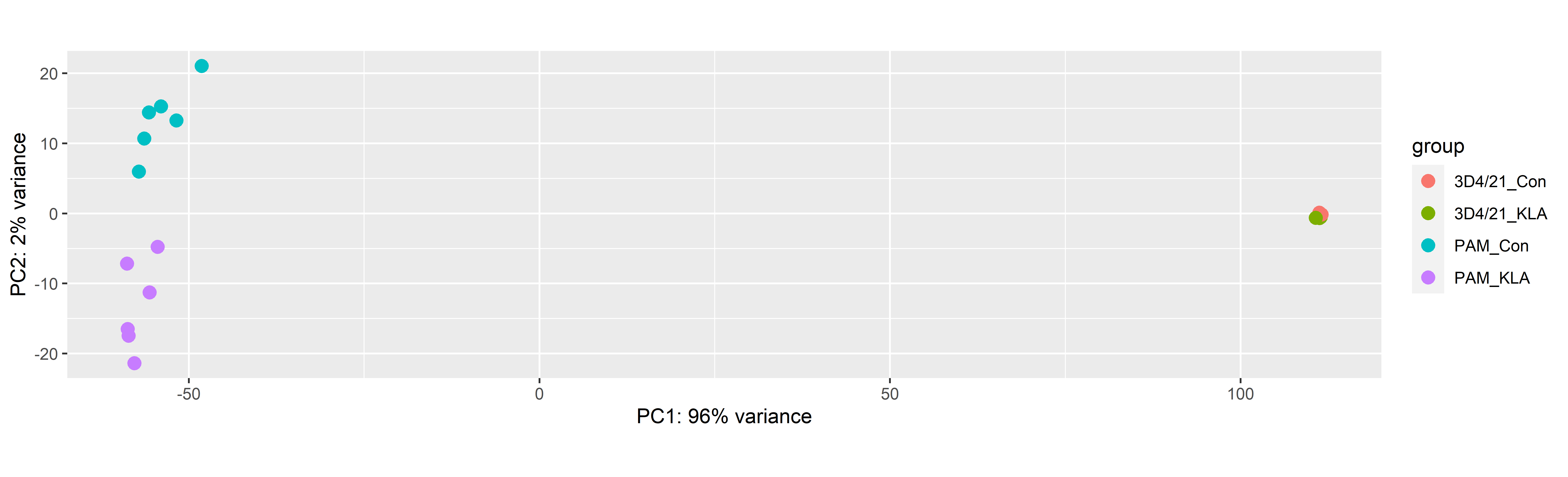


**Additional file 9. Principal component analysis of 3D4/21 cells and of primary porcine alveolar macrophages (PAM) based on transcriptome data obtained in unstimulated conditions (Con) or after 24h stimulation using 100 ng/mL Kdo2-Lipid A (KLA).**
